# More Common Than You Think: Common Variable Immune Deficiency

**DOI:** 10.1155/2013/153767

**Published:** 2013-12-05

**Authors:** Abimbola Aderinto, Vibav Mouli, Danielle F. Resar, Linda M. S. Resar

**Affiliations:** ^1^Department of Medicine, The Johns Hopkins University School of Medicine, Baltimore, MD 21205, USA; ^2^Hematology Division, The Johns Hopkins University School of Medicine, Baltimore, MD 21205, USA; ^3^Department of Oncology, The Johns Hopkins University School of Medicine, Baltimore, MD 21205, USA; ^4^The Institute for Cellular Engineering, The Johns Hopkins University School of Medicine, Baltimore, MD 21205, USA; ^5^The Johns Hopkins University School of Medicine, Ross Research Building, Room 1025, 720 Rutland Avenue, Baltimore, MD 21205, USA

## Abstract

We report a challenging case of a 16-year-old male who presented with thrombocytopenia and eluded a definitive diagnosis for over 2 years. He was initially diagnosed with a viral illness, although he later developed adenopathy and splenomegaly. An evaluation by an oncologist was unrevealing. He worked on a farm with livestock exposure and was later diagnosed with an atypical, zoonotic infection. Despite appropriate antibiotic therapy, the thrombocytopenia and splenomegaly persisted. Further evaluation revealed that he has a relatively common immunologic disorder. He is currently doing well on appropriate therapy for this disorder.

## 1. Introduction

We report a diagnostic dilemma involving a 16-year-old male who presented with thrombocytopenia and nonspecific symptoms, including malaise and fever. He was diagnosed with a viral illness, although the thrombocytopenia persisted and he was found to have adenopathy and splenomegaly. He was ultimately diagnosed with an unusual infection acquired from livestock exposure. Although he was treated with appropriate antibiotics, the thrombocytopenia and splenomegaly persisted. An immunologic evaluation was ultimately performed and he was diagnosed more than two years after presentation. He was placed on appropriate therapy and is currently doing well. The protean manifestations of this disorder often result in a delayed diagnosis by several years and are reviewed in detail here. This disorder is more common than you think.

## 2. Case Presentation

A 16-year-old male presented with fever, malaise, and decreased energy for 3 days. He was diagnosed with a viral illness, although symptoms persisted. He developed bruising and lost 50 pounds over the ensuing 3 months. Physical examination showed an exudative pharyngitis. A throat culture was unremarkable and blood counts showed the following: hemoglobin 16 g/dL, white blood cell (WBC) count 3,400/mm^3^, and platelet count 80,000/mm^3^; a differential was not obtained.

Four months later, he was evaluated by an oncologist who noted axillary and inguinal adenopathy. The abdomen was obese and hepatosplenomegaly was not appreciated. Blood counts showed the following: hemoglobin 15.5 g/dL, platelet count 66,000/mm^3^ and WBC 2,900/mm^3^ with 54% neutrophils, 32% lymphocytes, 9% monocytes, and 5% eosinophils. There were atypical, reactive-appearing lymphocytes on the peripheral blood smear. Computed tomography (CT) of the chest, pelvis, and abdomen showed splenomegaly (19 cm) and diffuse abdominal lymphadenopathy. A biopsy of the left axillary node revealed reactive hyperplasia. Bone marrow aspirate and biopsy showed mild hypocellularity with normal trilineage hematopoiesis and no evidence for malignancy. Flow cytometry of the lymph node and bone marrow showed no abnormalities.

The malaise persisted and the patient was evaluated by an infectious disease specialist 8 months after presentation. Physical examination was unremarkable; the adenopathy had resolved. Thrombocytopenia and leukopenia persisted (platelet count 73,000/mm^3^; WBC count 3,200/mm^3^). Serology studies for CMV, EBV, HIV, and *Bartonella* were all negative. Because he worked on a farm with exposure to livestock, titers for brucellosis were sent and were negative. However, titers for *Coxiella burnetii* were consistent with an acute Q fever infection (phase I IgG Ab <1 : 16, phase II IgG 1 : 256). He was started on doxycycline (100 mg twice daily). A transthoracic echocardiogram showed possible vegetation, although a transesophageal echocardiogram was normal. Thrombocytopenia and leukopenia persisted and repeat imaging studies 6 and 12 months after presentation showed persistent intra-abdominal adenopathy and splenomegaly. He was therefore treated with doxycycline for 2 years after which there was no detectable *Coxiella *DNA by polymerase chain reaction. Given the persistent cytopenias, adenopathy, and splenomegaly for >2 years, the patient was referred to our hematology clinic. Past medical history was notable for occasional atopic dermatitis and tonsillectomy at age 3 for recurrent pharyngitis. Family history was notable for a paternal uncle who died from metastatic colon cancer. There was no family history of other malignancies or unusual infections.

**Figure 1:**
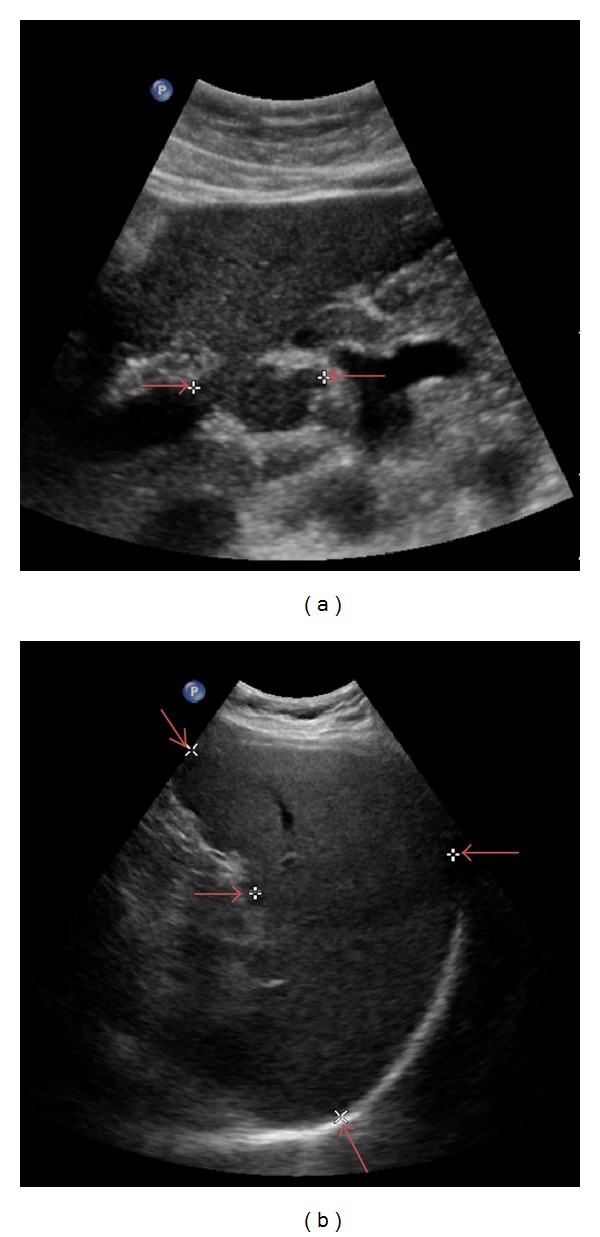
Abdominal ultrasound demonstrating lymphadenopathy and splenomegaly. (a) The lymph node diameter (3.1 cm) is demarcated by the arrows. (b) The spleen dimensions (18.3 cm × 9.3 cm) are demarcated by the arrows.

The patient appeared well and was afebrile with no appreciable adenopathy, organomegaly, or cutaneous bleeding. Blood counts showed the following: hemoglobin 15.4 g/dL, platelet count 100,000/mm^3^, and WBC count 4,077/mm^3^ with 25% lymphocytes, 8% monocytes, 63% neutrophils, and 4% eosinophils. The peripheral blood smear showed occasional, atypical, reactive-appearing lymphocytes. Peripheral blood flow cytometry showed no clonal abnormalities. Abdominal ultrasound showed persistent splenomegaly and adenopathy (Figure 1).

Although thrombocytopenia and splenomegaly can be associated with Q fever (Table 1) [1–6], the persistent cytopenias, adenopathy, and splenomegaly are distinctly unusual after 2 years of appropriate antibiotic therapy. Lymph node and bone marrow biopsies showed no evidence for malignancy. Thrombocytopenia can also occur with antiplatelet antibodies and immune-mediated thrombocytopenia purpura (ITP), although this is not typically associated with splenomegaly or leukopenia. The constellation of splenomegaly, thrombocytopenia, and leukopenia can also be caused by other immunological disorders. Thus, the patient's immunologic function was evaluated further and total quantitative immunoglobulin levels showed a profound deficiency (IgG 184 mg/dL (normal 751–1560), IgA < 7 mg/dL (normal 82–453), and IgM of 7 mg/dL (normal 46–304)). Following immunization to pneumococcus and tetanus, he had no detectable IgG antibodies to these vaccines. The inability to produce sufficient antibodies together with splenomegaly, thrombocytopenia, leukopenia, and hypogammaglobulinemia is consistent with common variable immune deficiency (CVID).

Our patient remains well on standard CVID therapy with weekly infusions of subcutaneous gamma globulin. His platelet count has remained stable (>100,000/mm^3^) and he has not had any significant infections.

## 3. Discussion

CVID is the most common primary immunodeficiency disorder [7–13]. CVID is characterized by the impaired secretion of antibodies (hypogammaglobulinemia; Table 2) and affects 1/10,000 to 1/100,000 individuals. The onset of symptoms typically occurs after puberty, but before 30 years [7–11]. Defective B-cell differentiation with inadequate secretion of immunoglobulins is the hallmark. Most patients experience a variety of complications, including respiratory tract infections, gastrointestinal symptoms, autoimmune disorders, malignancies, and viral infections. CVID is a diagnosis of exclusion with the following criteria: (1) age ≥4 years, (2) profound hypogammaglobulinemia, (3) failure to generate antibodies after exposure to two or more protein antigens, and (4) exclusion of other causes of hypogammaglobulinemia. The average period between onset of symptoms and diagnosis is six to eight years [7]. Despite the deficiency in antibody production, autoimmune diseases (immune-mediated thrombocytopenia, hemolytic anemia) are relatively common, and cytopenias occur in ~12% of patients [7–13]. Nonmalignant lymphoproliferative diseases are also common and manifested by lymphadenopathy and splenomegaly. Lymphoid hyperplasia can be difficult to distinguish from lymphoma, although most cases are benign [7, 8, 12]. Nonetheless, patients have an increased risk of lymphoid malignancies, particularly lymphoma (~5%) [8, 10, 11, 13]. Epidemiologic studies suggest an autosomal recessive mode of inheritance, although there are rare families that appear to have an autosomal dominant inheritance pattern. About 10–15% of CVID patients have disease-specific alleles and 90% of these appear to arise as *de novo* germline mutations [7, 9, 10]. Disease-specific alleles have been identified in *CD19*, *ICOS*, *TNFRSF13B*, *TNFRSF13C*, *CD20, CD81*, and *Msh5 *genes (Table 3) [7, 10].

Like CVID, Q fever can be a challenging diagnosis because of nonspecific symptoms (Table 1) [1–6]. Q fever is a zoonotic disease caused by *Coxiella burnetii* and transmitted to humans following contact with livestock (goats, cattle, and sheep) and inhalation of aerosolized particles. Q fever has a widespread geographic distribution worldwide with sporadic outbreaks. Since Q fever became a reportable disease in the USA in 1999, its incidence has been increasing [1, 2, 4]. Diagnosis relies on serologic findings, characterized by antibodies to phase I and phase II antigens that result from lipopolysaccharide modifications. The most common manifestations include fever, headaches, myalgias, malaise, and hepatitis [1–6]. Thrombocytopenia occurs in ~35% of the cases but is more commonly associated with endocarditis or pregnancy [3]. Treatment is typically doxycycline for two weeks. In chronic infections (lasting >6 months), IgG phase I antibodies predominate and are often >1 : 800 [5]. Endocarditis is the most common complication of chronic infection [2]. An echocardiograph is recommended for all patients, and those with vegetations should be treated for ≥12 months [2]. Serology studies are monitored at 3 and 6 months after the initial diagnosis to determine chronicity. Chronic Q fever is treated with doxycycline and hydroxychloroquine (≥18 months) [5]. Alternatively, doxycycline and ofloxacin can be used, but this typically requires longer durations of treatment (≥3 years) [5].

This case highlights the protean and often elusive manifestations of CVID, which led to a delayed diagnosis in our patient despite characteristic symptoms. The unusual infection (*Coxiella burnetii*) made the diagnosis challenging because chronic infections with Q fever can occur and mimic CVID. Nonetheless, the diagnosis of CVID should be considered in all patients presenting with cytopenias, splenomegaly, and atypical infections because therapy with gamma globulin is effective, as demonstrated by this interesting patient.

## Figures and Tables

**Table 1:** Clinical manifestations of Q fever [1–6].

Symptoms	Karagiannis et al., 2009 [1]	Sampere et al., 2003 [2]	Raoult et al., 2000 [3]	Domingo et al., 1999 [4]	Tselentis et al., 1995 [5]	Tissot Dupontet al., 1992 [6]	Average (*n* = 2,000)
	*n* = 73	*n* = 66	*n* = 1383	*n* = 63	*n* = 98	*n* = 323	
Fever	25%	84%	91%	100%	92%	82%	87%
Hepatitis		33%	62%	48%	52%		60%
Malaise	39%	33%	62%	48%	52%	53%	58%
Headache	41%	42%	51%	56%			50%
Chills		29%		59%			44%
Pulmonary involvement	22%	56%	34%	41%	89%	38%	38%
Myalgia/arthralgia	9%	41%	37%	46%			36%
Thrombocytopenia			35%				35%
Cough	37%	27%		35%			33%
Hepatomegaly		24%		54%	22%		31%
GI symptoms		27%		19%	16%		18%
Splenomegaly				22%	11%		16%
Weight loss	11%						11%
Rash			11%	2%	2%	21%	12%
Lymphadenopathy					7%		7%
Palpitations			4%				4%
Neurological symptoms			1%		12%	9%	3%
Jaundice	2%						2%
Myocarditis			1%				1%
Pericarditis			1%				1%

**Table 2:** Clinical manifestations of CVID [8–13].

Symptoms	Chapel et al., 2008 [10]	Oksenhendler et al., 2008 [11]	Quinti et al., 2007 [12]	Cunningham-Rundles and Bodian, 1999 [9]	Hermaszewski and Webster, 1993 [8]	Cunningham-Rundles, 1989 [13]	Average
	*n* = 334	*n* = 252	*n* = 224	*n* = 248	*n* = 240	*n* = 103	
Sinusitis, otitis media, bronchitis, and mastoiditis		69%	86%	98%	36%	100%	75%
Pneumonia		58%	49%	79%	63%	87%	69%
Bronchiectasis	25%	37%			18%		27%
Splenomegaly	30%	38%			13%		27%
Liver disease		17%					17%
Diarrhea		23%	23%	6%	12%		16%
Lymphadenopathy	15%				5%		11%
Urogenital infection			7%		7%		7%
Granulomatous disease	8%	14%	3%	8%	1%		7%
Thrombocytopenia	7%		6%	6%	3%	6%	7%
Non-Hodgkin's lymphoma	3%		2%	8%		8%	5%
Herpes/varicella				4%	7%	2%	5%
Hepatomegaly	9%				1%		5%
Hemolytic anemia	4%		3%	5%	3%	5%	4%
Pernicious anemia	9%			1%	2%	1%	4%
Hepatitis	2%			7%	1%	12%	4%
Meningitis/encephalitis		8%	1%	1%	7%	4%	4%
Sepsis		13%	1%	1%	2%		4%
Malaise					3%		3%
Crohn's disease	2%			4%		1%	3%
Vitiligo	5%		2%		2%		3%
Atopic dermatitis					2%		2%
Osteomyelitis				1%	3%	1%	2%
Autoimmune neutropenia	1%		3%	1%			2%
Stomach cancer			2%	1%		2%	2%
Septic arthritis			2%	1%	4%	1%	2%
Psoriasis	2%				1%		1%
Oral candidiasis			2%	1%	1%		1%
Parotitis				1%		1%	1%

**Table 3:** Genes implicated in CVID [7, 10].

Gene symbol	Protein encoded	Protein function	Chromosomal location	Estimated % CVID patients	Reference
*CD19 *	CD19	Protein component of the B-cell receptor complex	16p	<1%	Chapel et al., 2008 [10]

*ICOS *	Inducible costimulator of activated T-cells	Expressed on activated T-cells; facilitates cooperation between B and T-cells	2q	2%	Cunningham-Rundles, 2010 [7]

*TNFRSF13B *	Transmembrane activator and calcium-modulator and cyclophilin ligand (TACI)	Induces activation of transcription factors (NFAT, AP1, and NF-kappaB) and plays a crucial role in humoral immunity by interacting with a TNF ligand	17p	8%	Cunningham-Rundles, 2010 [7]

*TNFRSF13C *	BAFF-R, B-cell activating factor of the TNF family receptor	B-cell receptor specific for B-cell-activating factor (BAFF) which enhances B-cell survival	22q	Unknown	Chapel et al., 2008 [10]

*Msh5 *	MSH5	Involved in DNA mismatch repair or meiotic recombination processes	6p	12%	Chapel et al., 2008 [10]

*MS4A1 (CD20) *	MS4A1	Encodes a B-lymphocyte surface molecule involved in development and differentiation of B-cells into plasma cells	11q12-q13	<1%	Cunningham-Rundles, 2010 [7]

*CD81 *	CD81 (TAPA1)	Mediates signal transduction events involved in regulating cell development, growth, and motility	11p15.5	<1%	Cunningham-Rundles, 2010 [7]
